# STL2/386: HONselect: A new assisted-search facility for health information and resources integrating heterogeneous databases

**DOI:** 10.2196/jmir.1.suppl1.e106

**Published:** 1999-09-19

**Authors:** C Boyer, V Baujard, T Nater, JR Scherrer, RD Appel

**Affiliations:** ^1^Health On the Net Foundation, GenevaSwitzerland

**Keywords:** Integrated System, Search Engine, MEDLINE

## Abstract

**Introduction:**

Users looking for medical and health information today can investigate a widening assortment of sources. A full package of information on a specific subject can include descriptions of a disease or a condition, relevant articles from scientific journals, articles from a variety of popular newsletters, pages from different Web sites, links to support groups, patient advocates, conferences, continuing medical education, still and moving images and much more. However, finding and collating all this material is time-consuming, for the user must first painstakingly compile it from disparate sources. In response to growing user demand, HON has developed a new integrated search tool, HONselect, that helps meet the evolving needs of all users and is now available on the HON Web site

**Methods:**

The HONselect tool integrates the Medical Subject Headings (MeSH) terminology thesaurus, MEDLINE for scientific publication abstracts, HON's own MedHunt search engine for Web resources, the Newspage service for daily news and HON's Media Gallery for medical images. HONselect not only integrates the user's search for Internet information types and databases, it also offers a selection of resources available in each database. The National Library of Medicine (NLM) pioneered the concept with MEDLINEPlus which consists of a complementary resources selection such as Web sites and bibliographical references dedicated to patients

**Results:**

The HONselect tool enables the user to search different databases and to centralise the results in one interface as well as assist in finding the appropriate medical terms or MeSH headings by browsing or searching the MeSH hierarchical structure. But HONselect does more than add value to MEDLINE Plus. It, is a new concept which gives users the possibility of learning more about a given condition or disease in a scientifically structured fashion. in French and English, HONselect permits searching for medical words in natural language and finding the related scientific term, synonyms and definitions as well as their position in the hierarchical MeSH structure with both broader and narrower concepts and terms .

**Discussion:**

The Internet has always provided different types of information on a number of different subjects. This fact is particularly obvious in the medical domain. Until now, persons looking for details on a specific disease on the Web will first use a search engine that is either general or specialised in the medical domain. They will then select the Web site that appears best suited to their needs. But finding news, scientific papers or images usually means extending the search to further databases . HONselect is the solution (http://www.hon.ch/MeSH2/)

